# Anthocyanin-Rich Sour Cherry Extract Attenuates the Lipopolysaccharide-Induced Endothelial Inflammatory Response

**DOI:** 10.3390/molecules24193427

**Published:** 2019-09-21

**Authors:** Attila Biro, Arnold Markovich, Judit Rita Homoki, Erzsébet Szőllősi, Csaba Hegedűs, Szabolcs Tarapcsák, János Lukács, László Stündl, Judit Remenyik

**Affiliations:** 1 Institute of Animal Science, Biotechnology and Nature Conservation, Institute of Food Technology, University of Debrecen, H-4032 Debrecen, Hungary; biro.attila@agr.unideb.hu (A.B.); arnoldmarkovich@gmail.com (A.M.); homoki.judit@agr.unideb.hu (J.R.H.); szzsoka83@gmail.com (E.S.); stundl@agr.unideb.hu (L.S.); 2 Department of Pharmacology and Pharmacotherapy, University of Debrecen, H-4032 Debrecen, Hungary; csaba.hegedus.1983@gmail.com; 3 Department of Biophysics and Cell Biology, Faculty of Medicine, University of Debrecen, H-4032 Debrecen, Hungary; tarapcsakszabolcs@gmail.com; 4 Department of Obstetrics and Gynaecology, University of Debrecen, H-4032 Debrecen, Hungary; lukacs.janos@med.unideb.hu

**Keywords:** HUVECs, anthocyanins, sour cherry, inflammatory, cytokines, endothelial dysfunction

## Abstract

The anthocyanin content of Hungarian sour cherry is remarkable based on our preliminary investigations. Nutraceutical and pharmaceutical effects of anthocyanins have been extensively studied. The objective of this work was to investigate the the effect of purified sour cherry extract using human umbilical cord vein endothelial cells (HUVECs) as the inflammatory model. HUVECs were isolated by enzymatic digestion and characterized by flow cytometry. The optimal concentration range of sour cherry extract was selected based on MTT, apoptosis, and necrosis assays. Cells were divided into three groups, incubating with M199 medium as control, or with lipopolysaccharide (LPS) or with LPS plus anthocyanin extract (ACE). The effect of sour cherry extract on oxidative stress, pro-inflammatory factors, and arachidonic pathway was investigated. An amount of 50 μg/mL ACE (ACE_50_) was able to increase the level of glutathione and decrease the ROS, thereby improving the unbalanced redox status in inflammation. ACE_50_ lowered pro-inflammatory cytokine levels including Interleukin-6 (IL-6), regulated on activation, normal T cell expressed and secreted (RANTES), granulocyte-macrophage colony-stimulating factor (GM-CSF), and tumor necrosis factor alpha (TNF-α). ACE_50_ affected the arachidonic acid pathway by reducing the LPS-induced enzyme expression (cyclooxygenase-1, cyclooxygenase-2, and prostacyclin synthase). The extract under investigation seems to have a pleiotropic effect including anti-oxidative, anti-inflammatory, hemostatic, and vasoactive effects. Our results indicate that purified sour cherry extract could reduce the LPS-induced inflammatory response, thereby improving endothelial dysfunction.

## 1. Introduction

Polyphenol compounds including flavonoids are secondary metabolic products of plants that play a protective role against different stress effects. Anthocyanins are a subgroup of flavonoids. Regarding chemical structure, they have a positive charge at the oxygen atom of the C-ring of the basic flavonoid structure. Anthocyanins are commonly found in flowers and fruits, and some of these plants have been traditionally used as colorants and food [1]. The main sources of anthocyanins are red berry fruits, red grapes, red wine, crops (e.g., purple maize), and different red cabbage varieties [2]. Current research trends clearly indicate that natural products are one of the most important sources of new drugs [3]. Anthocyanins are also used in traditional medicine for the treatment of various diseases. In recent decades, anthocyanin-related research has proved its beneficial health effects [4]. Until the beginning of the century, anthocyanins appeared in scientific reports as chemical structures with a high antioxidant capacity. However, today we know that apart from their antioxidant capacity they can modulate several signal transduction pathways [5]. Anthocyanins possess anti-inflammatory [6], antimicrobial [7], anticancer [8], antidiabetic [9], and anti-obesity effects [10], as well as the notable prevention impact on cardiovascular diseases (CVDs) [11]. Furthermore, anthocyanins can inhibit human salivary α-amylase and the growth of *Streptococcus mutans* [12].

It is well known that anthocyanins reach maximum blood concentration within 3 h. This fast process is due to rapid absorption by the stomach [13]. Then, anthocyanins eliminate readily from the plasma by liver degradation [14]. The biological availability is <2% [15]. The anthocyanin level in plasma seems to be high enough to perform its biological activity, especially for those specific targets such as endothelial cells in veins, where anthocyanins significantly modulate intracellular signal transduction pathways and gene control activities [16].

Many studies have focused on their defensive role in inflammation [17]. A randomized control trial also showed that the level of plasma IL-1β of hypercholesterolemic patients who consumed an anthocyanin mixture (320 mg/day) for six months was substantially lower compared to placebo [18]. An in vivo study indicated that lipopolysaccharide (LPS)-induced mice fed with 10% blueberries had attenuated expression of protein and mRNA of tumor necrosis factor alpha (TNF-α) and interleukin-6 (IL-6) in the blood serum compared to the control [19]. An in vitro study demonstrated decreasing IFN-α-induced (100 ng/mL) expressions of MCP-1, IL-6, and TNF-α in human monocytic THP-1 cells due to a treatment of 10 mg/mL anthocyanin-containing bilberry extract [20]. Another in vitro study highlighted the protective effect of purified sour cherry anthocyanin extract on cytokine-induced inflammatory caco-2 monolayers [21].

The investigated Hungarian sour cherry varieties synthetize anthocyanins in high concentration. They produce selective cyanidin glycosides, the main component being cyanidin-3-*O*-rutinoside [22]. Other research has shown a wide range of polyphenolic compounds of sour cherry extract identified by mass spectrometer coupled to liquid chromatography (LC-MS). That study also clarified that the main anthocyanin components of sour cherry are cyanidin-3-rutinoside, cyanidin-3-*O*-glucoside, and cyanidin-3-*O*-glucosyl-rutinoside [23]. In our present study, the effect of sour cherry anthocyanin extract (ACE) was investigated in an inflammatory model. Inflammation is a biological response of body tissues to harmful stimuli, such as pathogens, damaged cells, or irritants, by a very complex instrument in which reactive oxygen species (ROS), cytokine burst, paracrine agents, balance of vasodilatation and vasoconstriction compounds, and balance of hemostasis play significant roles [24]. Because human umbilical cord vein endothelial cells (HUVECs) have been extensively used to study the biology and pathobiology of various diseases including the inflammatory process [25], we applied HUVECs as an inflammatory model. Inflammatory response was induced by LPS, an endotoxin from the outer membrane of bacteria, which is routinely used to trigger inflammation.

## 2. Results

### 2.1. Flow Cytometric Measurements

**Figure 1:**
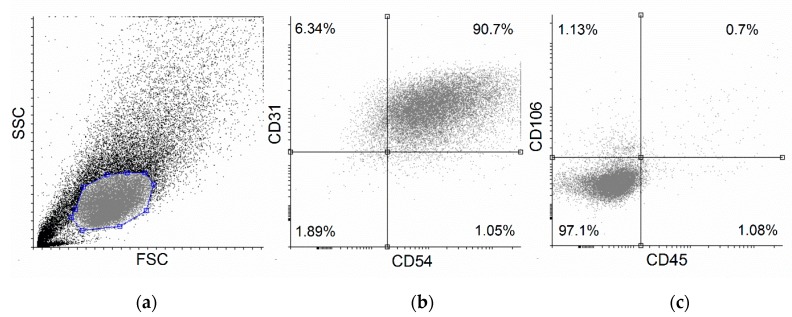
Flow cytometric analysis of human umbilical cord vein endothelial cells (HUVECs). Isolated HUVECs were checked for positive markers (**b**) CD54 and CD31 and negative marker proteins (**c**) CD45 and CD106 expression using specific antibodies. (**a**) Forward- and side-scatter plot and (**b**) dot-plots of HUVEC positive- and (**c**) negative-markers are shown. Representative graph of three independent experiments.

HUVECs were characterized to positive and negative marker expression using flow cytometry and antibodies against specific marker proteins ICAM-1 (intracellular adhesion molecule 1, CD54), PECAM-1 (Platelet endothelial cell adhesion molecule 1, CD31), PTPRC (protein tyrosine phosphatase receptor type C, CD45) and VCAM-1 (vascular cell adhesion molecule 1, CD106). As shown in Figure 1, isolated HUVECs showed high CD54 and CD31 positive marker expression levels (approximately 90% double positive cells), whereas approximately 97% of the cells did not express CD45 and CD106 markers (Figure 1), strongly suggesting the high accuracy and efficiency of cell isolation.

### 2.2. Main Compounds of Purified Sour Cherry Extract

**Figure 2:**
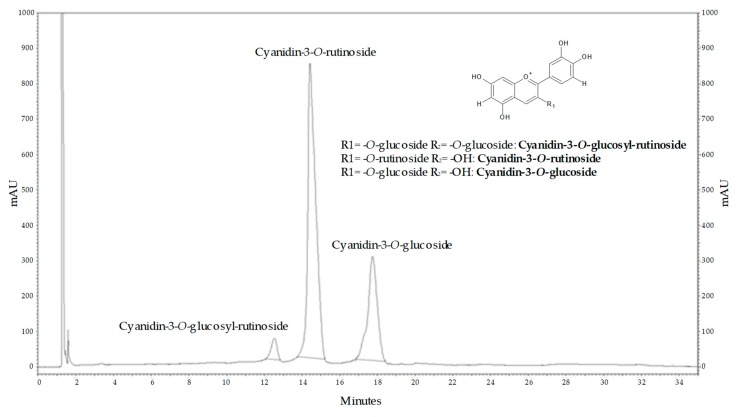
UHPLC chromatogram of sour cherry at 535 nm. Confirmed by standard.

Figure 2 shows the main components of the investigated sour cherry extract.

### 2.3. Seeking of Optimal ACE Concentration

#### 2.3.1. Up to 100 μg/mL, Survival Rate of HUVECs Was Not Altered by ACE Treatment

**Figure 3:**
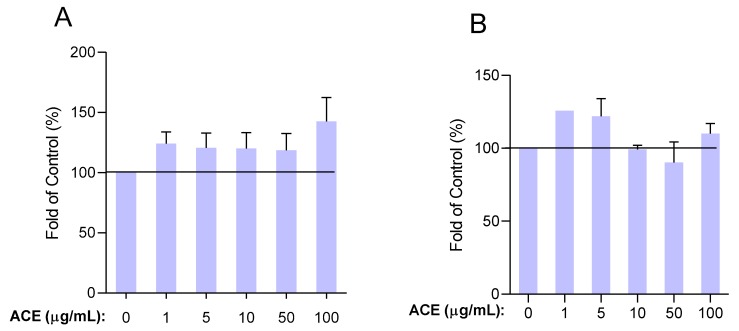
Viability of HUVECs was evaluated at (**A**) 24 h and (**B**) 48 h. Results are expressed in the percentage of the control (100% is represented by the solid lines). Data are expressed as the mean ± SEM of four individual experiments. ACE, anthocyanin extract.

**Figure 4:**
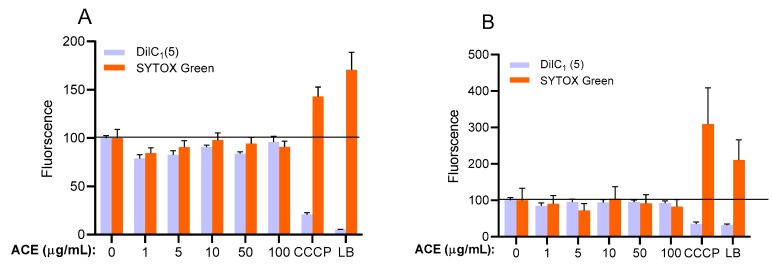
Combined fluorescent DilC_1_(5) and SYTOX Green labelling. To observe apoptotic and necrotic cell death, HUVECs were treated as indicated (**A**) 24 h or (**B**) 48 h. Results are expressed in the percentage of the control (100% is represented by the solid lines). Data are expressed as the mean ± SEM of four individual experiments. CCCP, carbonyl cyanide m-chlorophenyl hydrazone (positive control for apoptosis); LB, lysis buffer (positive control for necrosis); ACE, anthocyanin extract.

Prior to examining the effect of ACE on HUVECs, the effect of ACE on cell viability was evaluated. As shown in Figure 3, we found that ACE treatment at concentrations of 1, 5, 10, 50, and 100 μg/mL did not result in a significant change in viability of HUVECs. To confirm the promising results of the MTT assay, we also examined the potential apoptotic and necrotic effects of ACE using fluorescent labelling (DilC_1_(5) and SYTOX Green dyes) [26]. The results show that ACE did not induce apoptotic and necrotic events in this concentration range (Figure 4). These data suggest that the concentrations of ACE mentioned above can be used without the risk of any biologically relevant cytotoxic actions. According to our unpublished data, at higher concentrations of ACE (≥500 μg/mL) the viability of cells was substantially decreased and necrotic cell death was observed.

#### 2.3.2. Determination of Effective Antioxidant Concentrations

**Figure 5:**
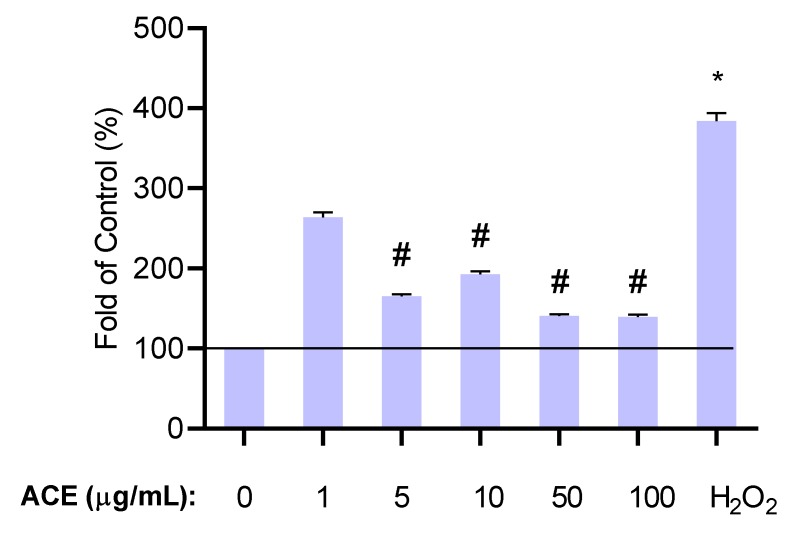
Effect of ACE on the level of reactive oxygen species (ROS) in various concentrations. * indicates the significant increase of ROS after H_2_O_2_ treatment compared to the control. # indicates the significant changes in ROS after H_2_O_2_ and ACE treatment compared to the H_2_O_2_ treatment without ACE. Results are expressed in the percentage of the control (100% is represented by the solid lines). Data are expressed as the mean ± SEM of four individual experiments. H_2_O_2_, hydrogen peroxide (positive control for oxidative stress); ACE, anthocyanin extract.

To select the appropriate concentration for further experiments, we also examined the antioxidant effect of ACE. We wanted to exclude the possibility that ACE has insufficient antioxidant capacity at low concentrations indicating biological inactivity. Cells were treated with H_2_O_2_ and levels of reactive oxygen species (ROS) were evaluated. Amounts of 50 and 100 μg/mL were the most effective in eliminating ROS (Figure 5). Since the maximum absorption of the main components (cyaniding-3-*O*-rutinoside) of the ACE in the blood is closer to 50 μg/mL, this concentration was selected (ACE_50_) and applied for further experiments [27].

### 2.4. ACE_50_ Inhibits LPS-Induced ROS in HUVECs

**Figure 6:**
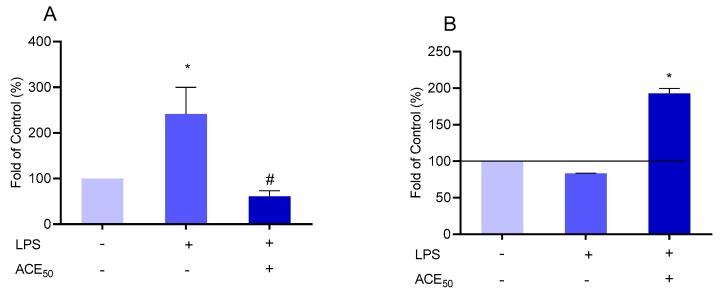
Effect of ACE_50_ on the level of ROS (**A**). * indicates the significant increase of ROS in lipopolysaccharide (LPS)-induced inflammation compared to the control. # indicates the significant changes in ROS in LPS-induced inflammation and ACE_50_ treatment compared to the level of ROS in LPS-induced inflammation without ACE_50_. Effect of ACE_50_ on the level of GSH (**B**). * indicates the significant increase of GSH after ACE_50_ treatment in LPS-induced inflammation compared to the control. Results are expressed in the percentage of the control (100% is represented by the solid lines). Data are expressed as the mean ± SEM of four individual experiments. ACE_50_, 50 μg/mL anthocyanin extract.

ROS production plays a pivotal role in inflammation [28]. It is well known that anthocyanins have excellent antioxidant properties. The effect of ACE_50_ on the level of ROS in HUVECs was evaluated (Figure 6A). LPS induced a considerable ROS production in the HUVECs. Incubating HUVECs with ACE_50_ led to a decreased amount of ROS. We also examined the level of glutathione (GSH), which is a good indicator of the antioxidant status [29] (Figure 6B). The level of GSH was significantly higher after ACE_50_ treatment.

These observations indicate that ACE_50_ can improve the imbalance of pro- and anti-oxidative agents causing the alleviation of oxidative stress.

### 2.5. ACE_50_ Decreases Secretion of Pro-Inflammatory Cytokines and Chemokines

**Figure 7:**
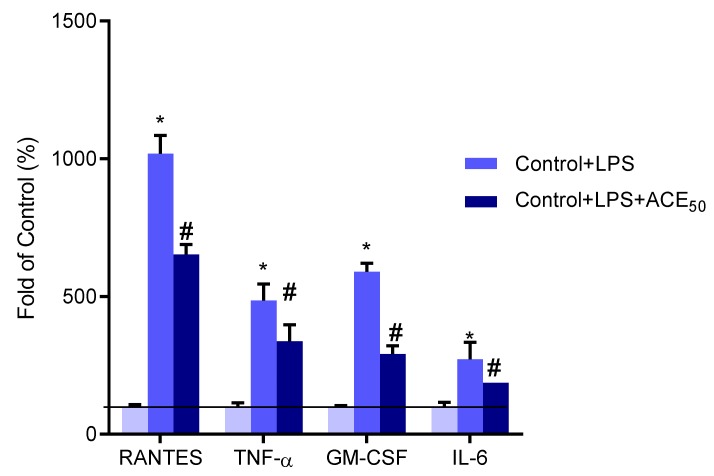
The level of evaluated cytokines and chemokines increased significantly compared to the control after LPS-treatment indicated by *. # indicates the significant changes in cytokines in LPS-induced inflammation and ACE_50_ treatment compared to the level of cytokines in LPS-induced inflammation without ACE_50_. Results are expressed in the percentage of the control (100% is represented by the solid lines). Data are expressed as the mean ± SEM of four individual experiments. ACE_50_, 50 μg/mL anthocyanin extract. Interleukin-6 (IL-6), regulated on activation, normal T cell expressed and secreted (RANTES), granulocyte-macrophage colony-stimulating factor (GM-CSF), and tumor necrosis factor alpha (TNF-α).

To determine the inhibitory effect of ACE_50_ on cytokine and chemokine secretion by HUVECs, IL-6, TNF-α, granulocyte-macrophage colony-stimulating factor (GM-CSF) and regulated on activation, normal T cell expressed and secreted (RANTES) levels were evaluated in supernatant after treatment with 100 ng/mL LPS in the presence or absence of ACE_50_. ACE_50_ was able to attenuate the level of the above-mentioned cytokines and chemokines (Figure 7). These observations confirm that sour cherry ACE_50_ can reduce inflammation on LPS-stimulated HUVECs by suppressing cytokine and chemokine release.

### 2.6. ACE_50_ Decreases Level of Tissue-Type Plasminogen Activator (tPA)

**Figure 8:**
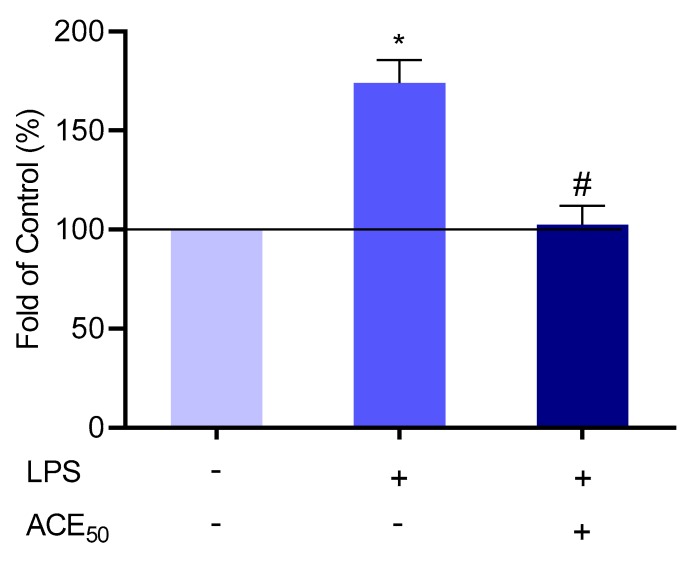
The level of tPA increased significantly compared to the control after LPS-treatment indicated by *. # indicates the significant changes in tPA in LPS-induced inflammation and ACE_50_ treatment compared to the level of tPA in LPS-induced inflammation without ACE_50_. Results are expressed in the percentage of the control (100% is represented by the solid lines). Data are expressed as the mean ± SEM of four individual experiments. ACE_50_, 50 μg/mL anthocyanin extract.

tPA has a dual function. As a serine protease, tPA plays a key role in the homeostasis of hemostasis and the extracellular matrix regulation. As a cytokine, tPA accomplishes multiple actions by binding to its membrane receptors and triggering intracellular signaling events [30]. tPA was investigated in the LPS-induced inflammatory model and assessed in supernatant after treatment with 100 ng/mL LPS in the presence or absence of ACE_50_. ACE_50_ had a similar effect on the level of tPA as on the above-mentioned cytokines. ACE_50_ was able to reduce the level of tPA in LPS-induced inflammation in HUVECs (Figure 8).

### 2.7. ACE_50_ Impact on Arachidonic Acid Pathway

**Figure 9:**
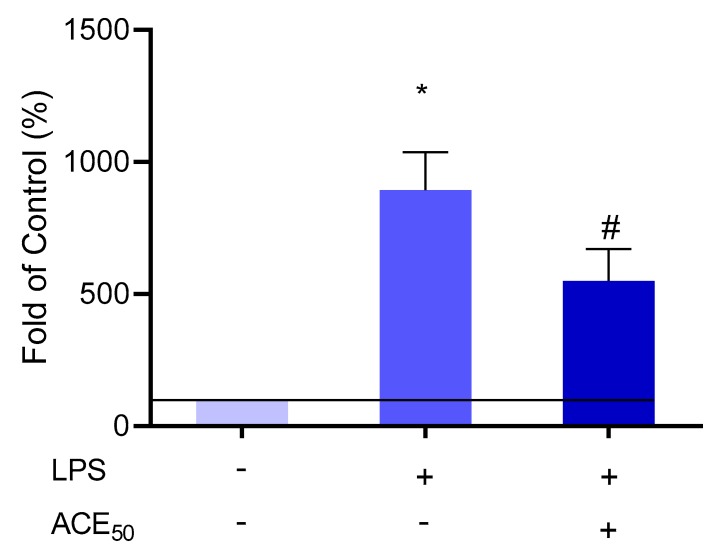
The level of PGI_2_ increased significantly compared to the control after LPS-treatment indicated by *. # indicates the significant changes in PGI_2_ in LPS-induced inflammation and ACE_50_ treatment compared to the level of PGI_2_ in LPS-induced inflammation without ACE_50_. Results are expressed in the percentage of the control (100% is represented by the solid lines). Data are expressed as the mean ± SEM of four individual experiments. ACE_50_, 50 μg/mL anthocyanin extract.

Regulatory elements of inflammation are mainly protein derivatives (cytokines, chemokines). In addition, arachidonic acid derivatives play an important role in the regional control of inflammation due to their very low half-life [31,32]. We investigated eicosanoids, which have both a vasoactive effect on smooth muscle cells and aggregative/anti-aggregative effects on platelets [32]. Since ACE_50_ can influence inflammatory response, we investigated the arachidonic acid pathway as well. We examined the level of thromboxane (TxA_2_) and prostacyclin (PGI_2_) in our inflammatory model. As shown in Figure 9, we found that ACE_50_ has a strong effect on the level of PGI_2_ (according to data not shown, the level of TxA_2_ was not influenced by ACE_50_).

**Figure 10:**
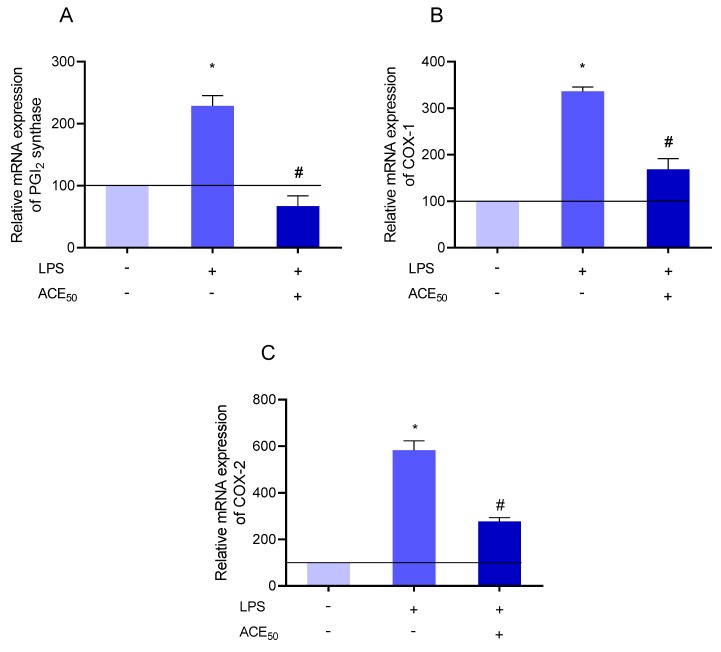
(**A**) The level of prostacyclin (PGI_2_) synthase mRNA increased significantly compared to the control after LPS-treatment indicated by *. # indicates the significant changes in PGI_2_ synthase mRNA in LPS-induced inflammation and ACE_50_ treatment compared to the level of PGI_2_ synthase mRNA in LPS-induced inflammation without ACE_50_. (**B**) The level of cyclooxygenase-1 (COX-1) mRNA increased significantly compared to the control after LPS-treatment indicated by *. # indicates the significant changes in COX-1 mRNA in LPS-induced inflammation and ACE_50_ treatment compared to the level of COX-1 mRNA in LPS-induced inflammation without ACE_50_. (**C**) The level of COX-2 mRNA increased significantly compared to the control after LPS-treatment indicated by *. # indicates the significant changes in COX-2 mRNA in LPS-induced inflammation and ACE_50_ treatment compared to the level of COX-2 mRNA in LPS-induced inflammation without ACE_50_. Results are expressed in the percentage of the control (100% is represented by the solid lines). Data are expressed as the mean ± SEM of four individual experiments. ACE_50_, 50 μg/mL anthocyanin extract.

Therefore, we investigated the expression of enzymes involved in prostacyclin synthesis by measuring their mRNA levels. The mRNA of cyclooxygenase-1 (COX-1), cyclooxygenase-2 (COX-2), and prostacyclin synthase (PGI_2_ synthase) was tested. It is well known that COX enzymes, with particular reference to COX-2, and PGI_2_ synthase are upregulated in inflammation [31]. In our experiments, we confirmed these facts. Furthermore, we found that ACE_50_ decreased the LPS-induced expression of COX-1, COX-2, and PGI_2_ synthase as determined by Q-PCR (Figure 10). The greatest mitigating effect was observed in the expression of PGI2 synthase (Figure 10A).

## 3. Discussion

Endothelium plays an important role in mediating inflammation, maintaining hemostasis, balancing vasodilatation and vasoconstriction, among other roles [33]. We investigated the effect of anthocyanin-rich sour cherry extract on LPS-induced inflammation in HUVECs in relation to the above. First, we assessed the optimal concentration of anthocyanin-rich sour cherry extract in our experiments. MTT, apoptosis, and necrosis assays were performed to seek the maximal non-harmful concentration. A reactive oxygen radical elimination ability test was carried out to seek the minimal effective antioxidant concentration. Based on these measurements, the amount of 50 μg/mL ACE (ACE_50_) was selected as the final concentration for further experiments.

Several studies have shown that anthocyanins are excellent antioxidant compounds [34]. Antioxidant properties may be mediated in several ways. On the one hand, anthocyanins as proton donors can directly eliminate reactive species. Therefore, we tested the effect of ACE_50_ on the formation of ROS in LPS-induced HUVECs. ACE_50_ was able to significantly reduce the formation of ROS. On the other hand, flavonoids can indirectly increase the expression of gene-encoding γ-glutamyl cysteine synthase, which determines the rate of glutathione synthesis [35,36]. We investigated the level of GSH and confirmed that ACE_50_ increases the level of GSH. These observations demonstrate that ACE_50_ can improve the imbalance of pro- and anti-oxidative agents, thereby alleviating oxidative stress.

Moreover, we examined the inflammatory response to LPS by measuring the secretion of chemokines and cytokines. IL-6 is a soluble mediator with a pleiotropic effect on inflammation, immune response, and hematopoiesis. It may have a central role in host defense mechanisms because of its effect on B-lymphocyte, T-lymphocyte, and hematopoietic stem cells, among others [37]. Tumor necrosis factor alpha (TNF-α) is an inflammatory cytokine produced by macrophages and monocytes during acute inflammation and is responsible for a diverse range of signaling events within cells [38]. Endothelial cells also produce chemokines that regulate eosinophil chemotaxis including RANTES [39]. Another chemokine, granulocyte-macrophage colony-stimulating factor (GM-CSF), is an important hematopoietic growth factor and immune modulator. GM-CSF also has profound effects on the functional activities of various circulating leukocytes [40]. Levels of IL-6, TNF-α, RANTES and GM-CSF were investigated in our experimental setup to evaluate the effect of ACE_50_ on inflammation. According to recently published articles, dietary anthocyanins can lower levels of these cytokines and chemokines in vitro and in vivo [17,41,42]. In accordance with the above, sour cherry-originated ACE_50_ decreased these pro-inflammatory cytokines and chemokines significantly. This suggests that ACE_50_ can modulate inflammatory response.

Considering the multifunctionality of the endothelium and the above-mentioned observations, we investigated the tPA, which serves as a cytokine and a hemostatic agent [30]. As a two-edged sword, tPA can be a promising target to alleviate inflammatory changes. We confirmed that sour cherry ACE_50_ decreases the level of tPA in LPS-induced HUVECs.

Furthermore, we investigated the effect of ACE_50_ on eicosanoids because arachidonic acid derivatives play a pivotal role in the local regulation of inflammation [43]. We found that ACE_50_ has a significant effect on PGI_2_ among eicosanoids but not on TxA_2_. To describe more accurately the mechanism of action, we tested the expression of COX-1, COX-2, and PGI_2_ synthase. The data suggest that the place of effect for the sour cherry ACE_50_ can found around the prostacyclin synthesis.

Since prostacyclin is a potent vasodilator, ACE_50_ may have a vasoactive effect. Further measurements are needed to confirm that hypothesis.

In summary, ACE_50_ has a pleiotropic effect in our inflammation model, including anti-oxidative, anti-inflammatory, hemostatic and vasoactive effects. With respect to mechanisms of action and possible clinical value, further investigations are needed since the sour cherry anthocyanin extract may have a therapeutic potential in endothelial dysfunction-associated diseases.

## 4. Materials and Methods

### 4.1. Materials

Purified sour cherry anthocyanin extract (ACE) was prepared by solid phase extraction procedure, as described earlier [22]. All other reagents were obtained from iBioTech Hungary Ltd. (Budapest, Hungary) and Nucleotest Bio Ltd. (Budapest, Hungary).

### 4.2. Methods

#### 4.2.1. Cell Culture Conditions

The human umbilical cord vein endothelial cells (HUVECs) were isolated from human umbilical cords. Umbilical cords were obtained from the Department of Obstetrics and Gynaecology, Clinical Centre, University of Debrecen, Debrecen, Hungary. The vein of the umbilical cord was cannulated and washed twice with Hank’s Balanced Salt Solution (HBSS). Digestion of HUVECs was performed with 22 U/100 mL collagenase solution incubating for 15 min at 37 °C. The cell pellet was suspended and transferred to a 75 cm^2^ flask. HUVECs were grown using M199 medium supplemented with 10% heat-inactivated FBS, 1% penicillin/streptomycin, 1% amphotericin B, 2 mM glutamine, and Endothelial Cell Growth Medium-2 (EGM-2) at 37 °C in a humidified incubator under 5% CO_2_. Media was changed every 48 h until cells reached 80% to 90% confluency. At confluency, cells were either subcultured or used for experiments. For all experiments, cells were growing at passage 1–4. Media as described above was used as control. To create an inflammatory model, LPS was added to M199 medium to a final concentration of 100 ng/mL. Cells were divided into three groups, 24 h incubating with M199 medium (Control), 24 h incubating with 100 ng/mL LPS (Control + LPS), and 24 h incubating with 100 ng/mL LPS plus 50 μg/mL ACE (Control + LPS + ACE_50_).

#### 4.2.2. Flow Cytometry Studies

For the identification of HUVECs, cells were incubated with four fluorescent dye-labeled antibodies for 30 min at room temperature in the dark. Approximately 100,000 cells were used for one measurement. Cell counting was performed using a Bürker chamber (Hirschmann Laboratory, Eberstadt, Germany). Amounts of 20 μL of fluorescein-isothiocyante (FITC)-labeled mouse anti-human CD31, 20 μL phycoerythrin (PE)-labeled anti-human CD54, 20 μL allophycocyanin (APC)-labeled mouse anti-human CD106, and 5 μL PerCP-Cy5.5 mouse anti-human CD45 were used for labelling. The measurements were carried out using a Becton Dickinson FACSAriaIII Cell Sorter (Becton Dickinson, Mountain View, CA, USA). FITC and PerCP-Cy5.5 were excited with a 488 nm laser (Becton Dickinson), and the emitted green light of FITC was detected using a 530/30 band-pass filter (Becton Dickinson), whereas the far-red fluorescence of PerCP-Cy5.5 was measured using a 635 dichroic mirror (Becton Dickinson) and 695/40 band-pass filter (Becton Dickinson). PE was excited by the 562 nm line of a solid state laser and the emitted light was detected applying a 595/50 nm band-pass filter. APC was excited with a 635 nm red laser and fluorescence intensity was detected using a 660/20 band-pass filter (Becton Dickinson). In all, 20,000 cells/sample were recorded and analyzed. Cytofluorimetric data were analyzed using FCS Express 4 Research Edition (De Novo Software, version 7, Glendale, CA, USA).

#### 4.2.3. Determination of Cellular Viability

Cell viability was determined by MTT assay measuring the conversion of tetrazolium salt to formazan crystals by mitochondrial dehydrogenases. Cells were seeded to 96-well plates at a density of 20,000 cells per well in quadruplicates and were treated with sour cherry extract of different concentrations (1, 5, 10, 50, and 100 μg/mL) for 24 and 48 h. Cells were then incubated with 0.5 mg/mL MTT solution for 3 h. Formazan crystals formed proportional to the number of viable cells. After dissolving them in isopropyl alcohol, absorbance was measured colorimetrically at 465 nm using a Clariostar microplate reader (BMG Labtech, Ortenberg, Germany). The results were expressed relative to 100% of the control group.

#### 4.2.4. Determination of Apoptosis

Decrease in the mitochondrial membrane potential is one of the earliest markers of apoptosis. Therefore, to estimate the process, the mitochondrial membrane potential of the HUVECs was determined using DilC_1_(5) dye. Cells (20,000 cells/well) were cultured in 96-well plates and treated as indicated in Section 4.2.4. After removal of the supernatants, the cells were incubated for 30 min with a DilC_1_(5) working solution (50 μL/well) and then washed with PBS, and the fluorescence of DilC_1_(5) was measured at 630 nm excitation and 670 nm emission wavelengths using a Clariostar microplate reader. The results were expressed relative to 100% of the control group.

#### 4.2.5. Determination of Necrosis

Necrotic processes were evaluated by SYTOX Green staining. Cells were cultured in 96-well plates, and treated as indicated in Section 4.2.4. Then, the supernatants were discarded, and the cells were incubated for 30 min with 1 μM SYTOX Green dye. After incubation, cells were washed with PBS, and the fluorescence of SYTOX Green was measured at 490 nm excitation and 520 nm emission wavelengths using a Clariostar microplate reader. The results were expressed relative to 100% of the control group.

#### 4.2.6. Measurement of ROS

The cells were treated in a 24-well plate and exposed to 100 μM 2′,7′-dichlorofluorescin diacetate (DCFDA) for 1 h at 37 °C to label intracellular ROS. After incubation, the cells were washed twice with PBS. Then, the labelled cells were monitoring every 6 h. Fluorescence intensity (excitation = 485 nm; emission = 530 nm) was measured using a microplate reader (Clariostar; BMG Labtech). The results were expressed relative to 100% of the control group.

#### 4.2.7. Measurement of GSH Level

The assay was performed according to the manufacturer′s instructions (Abcam Plc., Cambridge, UK) using a Glutathione Assay Kit (Abcam Plc.). The results were expressed relative to 100% of the control group.

#### 4.2.8. Luminex MagPlex Assay

HUVECs were treated with 100 ng/mL LPS for 24 h and the supernatant was collected and centrifuged for 10 min 10,000 r·min^−1^ and stored at −80 °C. The supernatant levels of IL-6, GM-CSF, TNF-α, and RANTES were determined using a MILLIPLEX MAP Human cytokine/chemokine Magnetic Bead Panel (HCYTOMAG-60K-09, EMD Millipore Corp., Billerica, MA, USA) according to the manufacturer′s instructions. The results were expressed relative to 100% of the control group.

#### 4.2.9. ELISA

HUVECs were treated with 100 ng/mL LPS for 24 h and the supernatants were collected and centrifuged for 10 min 10,000 r·min^−1^ and stored at −80 °C. The concentrations of PGI_2_ and tPA were measured in the cell supernatant with a competitive (PGI_2_) and sandwich (tPA) ELISA (Abcam Plc., Cambridge, UK) according to the manufacturer’s instructions. The results were expressed relative to 100% of the control group.

#### 4.2.10. Q-PCR

Q-PCR was performed on a Roche LightCycler 480 System (Roche, Basel, Switzerland) using the 5′ nuclease assay. Total RNA was isolated using Extrazole. 1 μg of total RNA was reverse-transcribed into cDNA using an UltraScript 2.0 cDNA Synthesis Kit (PCR Biosystems, London, UK). The amplification was performed using the 2× qPCRBIO Probe Mix No-ROX assay (PCR Biosystems, London, UK). As internal control, glyceraldehyde 3-phosphate dehydrogenase was determined. The results were expressed relative to 100% of the control group.

#### 4.2.11. Statistical Analysis

For multiple comparisons, results were analyzed by ANOVA followed by modified *t*-test for repeated measures according to Bonferroni’s method. Data were presented as mean ± SEM. *p* < 0.05 was considered statistically significant.

### 4.3. Ethics

The study was conducted in accordance with the Declaration of Helsinki, and the protocol was approved by the Ethics Committee of the University of Debrecen (registration number RKEB/IKEB 3712-2012).
